# Failure of the Anti-Inflammatory Parasitic Worm Product ES-62 to Provide Protection in Mouse Models of Type I Diabetes, Multiple Sclerosis, and Inflammatory Bowel Disease

**DOI:** 10.3390/molecules23102669

**Published:** 2018-10-17

**Authors:** James Doonan, David Thomas, Michelle H. Wong, Hazel J. Ramage, Lamyaa Al-Riyami, Felicity E. Lumb, Kara S. Bell, Karen J. Fairlie-Clarke, Colin J. Suckling, Kathrin S. Michelsen, Hui-Rong Jiang, Anne Cooke, Margaret M. Harnett, William Harnett

**Affiliations:** 1 Strathclyde Institute of Pharmacy and Biomedical Sciences, University of Strathclyde, Glasgow G4 0RE, UK; james.doonan@strath.ac.uk (J.D.); hazeljramage@gmail.com (H.J.R.); lamyaa.alriyami@gmail.com (L.A.-R.); felicity.lumb@strath.ac.uk (F.E.L.); karabell@hotmail.co.uk (K.S.B.); karenfairlieclarke@gmail.com (K.J.F.-C.); huirong.jiang@strath.ac.uk (H.-R.J.); 2 Department of Pathology, University of Cambridge, Cambridge CB2 1QP, UK; tdct2@cam.ac.uk (D.T.); ac416@cam.ac.uk (A.C.); 3 F. Widjaja Foundation Inflammatory Bowel and Immunobiology Research Institute, Department of Medicine, Cedars-Sinai Medical Center, Los Angeles, CA 90048, USA; Michelle.Wong@cshs.org (M.H.W.); Kathrin.Michelsen@cshs.org (K.S.M.); 4 Department of Pure & Applied Chemistry, University of Strathclyde, Glasgow G1 1XL, UK; c.j.suckling@strath.ac.uk; 5 Institute of Infection, Immunity and Inflammation, College of Medical, Veterinary and Life Sciences, University of Glasgow, Glasgow G12 8TA, UK; margaret.harnett@glasgow.ac.uk

**Keywords:** ES-62, helminth, inflammatory bowel disease, multiple sclerosis, nematode, type 1 diabetes

## Abstract

Parasitic helminths and their isolated secreted products show promise as novel treatments for allergic and autoimmune conditions in humans. Foremost amongst the secreted products is ES-62, a glycoprotein derived from *Acanthocheilonema viteae*, a filarial nematode parasite of gerbils, which is anti-inflammatory by virtue of covalently-attached phosphorylcholine (PC) moieties. ES-62 has been found to protect against disease in mouse models of rheumatoid arthritis, systemic lupus erythematosus, and airway hyper-responsiveness. Furthermore, novel PC-based synthetic small molecule analogues (SMAs) of ES-62 have recently been demonstrated to show similar anti-inflammatory properties to the parent molecule. In spite of these successes, we now show that ES-62 and its SMAs are unable to provide protection in mouse models of certain autoimmune conditions where other helminth species or their secreted products can prevent disease development, namely type I diabetes, multiple sclerosis and inflammatory bowel disease. We speculate on the reasons underlying ES-62’s failures in these conditions and how the negative data generated may help us to further understand ES-62’s mechanism of action.

## 1. Introduction

The success of parasitic helminths in evading detection and expulsion from their hosts is believed to be due to a range of strategies that the parasites employ to regulate host immune responses and to maintain homeostasis. Thus, active helminth infection and also immunomodulatory helminth-derived excretory/secretory (ES) products have been shown to induce a range of regulatory immune responses that can skew host immunity (reviewed in [1]). Furthermore, such responses appear to have knock-on effects, as epidemiological studies have shown that exposure early in life to helminth infections is negatively correlated with the development of allergy and autoimmunity, and that anthelminthic therapies can result in loss of helminth-induced immunoregulation (reviewed in [1]).

Amongst the most widely characterised helminth ES product is ES-62, which is produced by *Acanthocheilonema viteae* (reviewed in [2]). ES-62 is a tetrameric protein that is decorated with phosphorylcholine (PC) moieties covalently attached to *N*-type glycans. The PC is largely responsible for ES-62’s immunomodulatory actions which, via degradation of MyD88 and downstream transducers like PKC-α, can dampen inflammatory responses to pathogen-associated molecular patterns (PAMPS) such as lipopolysaccharide (LPS), as well as to certain antigens (reviewed in [2]). Such subversion of immune signalling dictates that ES-62 can be used to treat disease, both prophylactically and therapeutically, in a number of pre-clinical models of autoimmunity and allergy, including rheumatoid arthritis (RA; collagen-induced arthritis (CIA) (reviewed in [2])), systemic lupus erythematosus (SLE; MRL/Lpr mouse model [3]), and asthma (acute and chronic ovalbumin (OVA)-induced asthma models [4,5]). This has raised the idea of employing ES-62 as a novel therapy, but such thoughts are tempered by its likely immunogenicity and by the fact that its active moiety is a post-translational modification. Therefore, a library of synthetic non-immmunogenic, small molecule analogues (SMAs), based around the structure of the active PC-moiety, was designed and screened to find compounds mimicking ES-62’s immunomodulatory properties [6]. From this work, SMAs 11a and 12b were identified as possessing anti-inflammatory profiles matching ES-62’s properties, both in vitro and in pre-clinical models of RA, SLE and allergy [6,7,8,9,10,11,12]. However as parasitic helminths and their ES products have been shown to be protective in a wide range of mouse models of inflammatory conditions (reviewed in [13]), we decided to investigate the therapeutic potential of ES-62 and its SMAs in other pathologies. In particular, we employed the non-obese diabetic (NOD) mouse model of type 1 diabetes (T1D), the experimental autoimmune encephalomyelitis (EAE) model of multiple sclerosis (MS), and the dextran sodium sulphate (DSS)-induced and T cell transfer models of inflammatory bowel disease (IBD) in an attempt to gauge the range of ES-62’s therapeutic potential.

The NOD mouse spontaneously develops a Th1 response to islet antigens that results in inflammatory infiltration of pancreatic islets from 4–5 weeks of age, leading to β cell destruction, insulitis, and diabetes. There are a number of reports of parasitic worms, their extracts and products preventing diabetes development in NOD mice. Thus, short term treatment of the mice from four weeks of age with non-viable eggs or soluble extracts from the egg or worm of the parasitic trematode *Schistosoma mansoni* was found to be sufficient to prevent the development of diabetes and preserve β islet cells [14]. Roles for NKT cells [14] and regulatory T cells (Tregs) [15] have been put forward to explain these data. More recently, ES products from another trematode, *Fasciola hepatica*, were also reported to prevent the onset of diabetes, specifically by inducing M2 peritoneal macrophages that are capable of generating a potent Treg response [16]. With respect to nematodes, protection against T1D was demonstrated using live *Litomosoides sigmodontis* larvae or adult worms and also adult worm antigen (LsAg), acting via the generation of a Th2 immunological phenotype and the induction of a Treg response [17]. Together, these data suggest that pathogenic Th1 responses can be subverted by helminth-induced Th2 polarisation and immunoregulatory responses, to prevent diabetes in the NOD mouse.

In MS models, unlike in the NOD mouse where disease develops spontaneously, EAE is generated following immunisation with myelin oligodendrocyte glycoprotein (MOG) or myelin basic protein (MBP) in Complete Freunds Adjuvant (CFA) to induce a potent Th1/17 autoimmune response. This model is characterised by infiltration of the central nervous system (CNS) by inflammatory T cells and macrophages, which leads to rapid paralysis. Administration of *S. mansoni* eggs, *S. japonicum* soluble egg antigen (SEA) or live *F. hepatica* infection was each found to suppress EAE by inducing strong Th2 and Treg responses in the CNS that promoted IL-4 and reduced IFN-γ production [18,19,20]. Furthermore, live infection with the model gastrointestinal nematode *Heligmosomoides polygyrus* resulted in remission of active EAE disease [21] and transfer of regulatory B cells from the mesenteric lymph nodes of such infected mice could prevent disease onset in recipient mice [22].

Inflammatory bowel disease (IBD) is an umbrella term for a collection of diseases, including ulcerative colitis and Crohn’s disease that can be investigated using a variety of in vivo pre-clinical models, in which helminth infections or the use of their ES products have proven to be effective in reducing disease. For example, mirroring its effects in diabetes and EAE, *S. mansoni* SEA induced potent Th2 and Treg responses that protected animals from generating an exaggerated immune responses towards DSS-induced colitis [23]. Using this same DSS model, ES products from the hookworm *Ancylostoma caninum* protected against colitis by dampening Th1/17 responses, again via the promotion of Th2 immune responses [24].

Despite the different aetiologies/target organs involved in these autoimmune diseases, there are similar underlying mechanisms; each disease being driven by a potent Th1/17 response that results in the pathological destruction of tissue. Here, we demonstrate that despite our previous success in exploiting the ability of ES-62 and the SMAs to dampen such responses, we were unable to (i) decrease diabetes incidence in the NOD mouse model, (ii) prevent the onset of paralysis in the EAE model, and (iii) protect against colonic inflammation in chronic IBD models. In spite of this, by reflecting on the successes and failures of ES-62, we hope to further elucidate the immunoregulatory mechanisms of action of this helminth product.

## 2. Materials and Methods

### 2.1. ES-62 and SMAs

Highly purified ES-62 was prepared from spent culture medium of adult mixed-sex *A. viteae* by size exclusion centrifugation using endotoxin-free reagents and procedures, as described previously [25]: SMAs were synthesised as previously described [6].

### 2.2. Type I Diabetes Model

The NOD mouse was employed as a model of type I diabetes (T1D), and initial experiments were based on regimens where ES-62 had been successfully employed to prevent the development of autoimmunity [3], and where extracts of adult *Schistosoma mansoni*, or its eggs, had been in effective in preventing diabetes development [14]. BDC2.5 mice are T cell receptor transgenic mice expressing a T cell receptor for islet antigen on CD4^+^ T cells [26]. The study was carried out in accordance with the recommendations of the Animals (Scientific Procedures) act and was conducted under UK Home Office project license regulations (numbers PPL 80/1560 and PPL 80/2242) after approval by the Ethical Review Committees of the University of Cambridge. NOD mice and BDC2.5 NOD transgenic mice were bred and maintained under specific pathogen-free barrier conditions. Mice were housed in individually ventilated cages with standard ad libitum conditions.

Female NOD mice received intraperitoneal injections of 2 μg ES-62 in sterile PBS (*n* = 10 mice) or sterile PBS alone (*n* = 13 mice) once a week from four weeks until 12 weeks of age. Non-diabetic female NOD mice (9–10-week-old, *n* = 9 mice/group) were injected six times intraperitoneally with sterile PBS (Control) or 10 μg of ES-62 in sterile PBS over a two-week period. For the SMA experiment, 5-week-old female NOD mice (*n* = 10 mice/group) received intraperitoneal injections of 1 μg of SMA 11a or 12b twice a week for 10 weeks. The incidence of diabetes was determined by monitoring glycosuria using Diastix reagent strips (Bayer Diagnostics, Basingstoke, UK) and confirmed by a blood glucose measurement of >13.3 mM, using a Breeze2 blood glucose meter (Bayer).

Bone marrow-derived dendritic cells (DCs) were generated by culturing bone marrow cells flushed from NOD femurs and tibia, red cell lysed, re-suspended and cultured at 10^6^/mL in Iscove’s Modified Dulbecco’s Medium (IMDM) supplemented with 10% foetal calf serum (FCS), penicillin (100 U/mL), streptomycin (100 μg/mL), 2-mercaptoethanol (25 μM), and l-glutamine (2 mM) in the presence of 20 ng/mL recombinant murine granulocyte-macrophage colony-stimulating factor (GM-CSF; Preprotech, London, UK) and 10 ng/mL IL-4 (Preprotech, London, UK). Non-adherent cells were discarded at day 3 and cells re-suspended in the supplemented medium described above for a further 5–7 days before the non-adherent cells were used as DCs. DCs were washed and adjusted to 5 × 10^4^ cells/well in a flat bottomed, 96-well plate in 200 μL final volume. The cells were set up in triplicate cultures and incubated with 500 ng/mL LPS (*S. minnesota*, Sigma, Poole, UK) PBS and/or ES-62. The supernatants were collected at 48 or 72 h and analysed for cytokines by sandwich enzyme-linked immunosorbent assay (ELISA).

Four-week-old BDC2.5NOD mice were given two injections of 2 μg ES-62 (in PBS with PBS as a control) over the course of a week either by the intraperitoneal or subcutaneous route. Single cell suspensions of red blood cell lysed spleen cells were cultured in the presence of BDC2.5 peptide in the presence or absence of 1 or 2 μg ES-62. Supernatants were taken at 72 h and the presence of cytokines in supernatants was assessed by ELISA. The BDC2.5 peptide (RTRPLWVRME) was supplied by Cambridge Peptides, Cambridge, UK.

Analysis of cytokines produced by DCs or spleen cells was carried out using ELISA kits (R&D systems, Abingdon, UK) for TNF-α IL-12p40, IL-6, IL-10, IL-4, and IFN-γ. Protocols were followed according to the manufacturer’s instructions.

Single-cell suspensions were made from spleens of BDC2.5NOD mice incubated with ES-62 as described above for flow cytometry. Cells were washed and re-suspended in staining buffer (PBS containing 2% bovine serum albumin and 0.05% NaN_3_). Fc receptors were blocked using antibody 24G2 (a kind gift from Dr Nick Holmes, University of Cambridge, Cambridge, UK). Cells were stained with the following monoclonal antibodies directly conjugated to fluorochromes: anti-CD19-FITC, anti-CD4-PE, anti-CD4-PerCP, and anti-CD25-PE (BD Pharmingen, Wokingham, UK), and then washed and analysed using a FACScan flow cytometer and CELLQUEST software (Becton Dickinson Europe, Erembodegem, Belgium).

### 2.3. Experimental Autoimmune Encephalomyelitis

EAE was employed as a model of MS using naïve C57BL/6 mice that were bred and maintained in the Biological Procedure Unit at the University of Strathclyde. Mixed-sex animals (we find no effect of gender on disease incidence or severity) at 7–9-weeks old were used in the experiments, which were performed under the guidelines of the UK Animals (Scientific Procedures) Act 1986.

Mice were immunised subcutaneously on day 0 with 50 μL of 150 μg MOG_35–55_ peptide (ChinaPeptides Co. Ltd., Shanghai, China) emulsified in 50 μL of CFA (Sigma) supplemented with 400 μg *Mycobacterium tuberculosis* (BD Biosciences). In addition, each mouse received two injections of pertussis toxin (PTX; Tocris Bioscience, Bristol, UK) intraperitoneally (150 ng in 100 μL PBS) on day 0 and day 2. Mice received three doses of treatment with PBS (Control), 2 μg of ES-62, or 1 μg of SMAs 11a or 12b on Day-2, Day 0, and Day 2 by the sub-cutaneous route. EAE clinical symptoms were carefully monitored, and the clinical score was recorded based on the following evaluation system: 0: normal with no clinical sign; 1: complete loss of tail tone; 2: hind limb weakness; 3: hind limb paralysis; 4: forelimb involvement in addition to hind limb paralysis; 5: moribund.

### 2.4. Induction and Assessment of Chronic Colitis

DSS-induced chronic colitis was initiated by multi-cycle administration of DSS drinking water [27]. Female mice of eight weeks of age received 2.3% (*w*/*v*) DSS (40,000–50,000 MW; MP Biomedicals, Irvine, CA, USA) in drinking water on days 1–5, 8–12, 15–19, and 22–26. Mice were injected with PBS, ES-62 (2 μg/mouse), SMA 11a or SMA 12b (1 μg/mouse each) three times per week starting on day 1 of DSS treatment. Mice were checked daily for the development of colitis by monitoring body weight, gross rectal bleeding, and stool consistency, and were sacrificed at day 29. The cecum and colon were removed, tissues were fixed in 10% formalin in PBS, paraffin-embedded, and cross sections were stained with haematoxylin and eosin (H&E). Lamina propria mononuclear cells (LPMC) were isolated from the large intestine as previously described [28]. Single-cell suspensions of LPMC were stained with anti-CD4 antibodies for flow cytometry. To determine the percentage of cells expressing surface markers and the intensity of expression, samples were examined using a CyAn™ ADP flow cytometer (Dako Cytomation, Carpinteria, CA, USA) and analysed with the FlowJo (TreeStar, Ashland, OR, USA). Percentages of CD4^+^ cells were analysed on the gated lymphocyte population.

### 2.5. T Cell Transfer Model

Female C57BL/6 mice were used for donors, and male *Rag1*^−/−^ mice of the C57BL/6 background were used as recipients. Spleens were homogenised and the resulting cell suspension was passed through a 25-gauge needle. CD4^+^ T cells were negatively selected using the EasySep Mouse CD4^+^ T Cell Enrichment Kit (STEMCELL Technologies Inc., Vancouver, BC, Canada). Cells were labeled with anti-CD4, anti-CD25, and anti-CD45RB. Using the MoFlow cell sorter (Dako Cytomation, Carpinteria, CA, USA), CD4^+^ CD25^−^ CD45RB^high^ cells were purified by gating and sorting 40% of the highest fluorescing CD45RB cells. Each recipient mouse was injected i.p. with 0.5 × 10^6^ cells in sterile PBS. Mice were injected with PBS, 11a, or 12b (1 μg/mouse each) three times per week, starting on day 1 of T cell transfers. Animals were weighed and observed for signs of colitis over a 6- to 8-week period. Mice were sacrificed at the indicated time points. Large intestines, and caeca were collected, formalin-fixed, paraffin-embedded, and stained with H&E.

## 3. Results

### 3.1. Type I Diabetes

**Figure 1:**
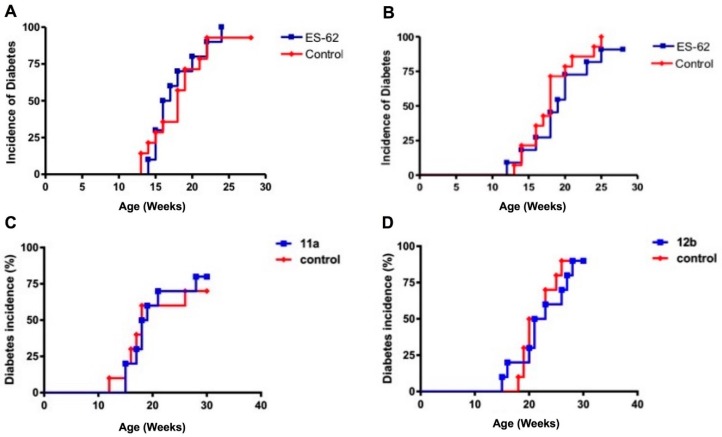
ES-62 and SMAs 11a and 12b do not protect against the development of type 1 diabetes. (**A**) Four-week-old female non-obese diabetic (NOD) mice were injected subcutaneously with 2 μg of ES-62 (*n* = 10) in 200 μL of sterile PBS once a week between four and 12 weeks of age. Control mice (*n* = 13) were injected with 200 μL of sterile PBS according to the same protocol. (**B**) 9–10 week-old female NOD mice were injected six times subcutaneously with sterile PBS (control) or 10 μg of ES-62 in sterile PBS over a two-week period (*n* = 9). Five-week-old female NOD mice received intraperitoneal injections of PBS (control) or 1 μg SMA 11a (**C**) or 12b (**D**) twice a week for 10 weeks (*n* = 10). The incidence of diabetes was monitored by regularly checking for the presence of glycosuria.

The NOD mouse spontaneously develops T1D, providing an animal model of the human autoimmune disease. Previous studies have shown that helminth infections or helminth-derived products are able to prevent the onset of diabetes in this mouse model (e.g., [14]). We therefore examined the ability of ES-62 (Figure 1A,B) or its SMAs (Figure 1C,D) to inhibit diabetes. We generally used time points that had shown efficacy in the previous helminth studies, and employed doses of ES-62/SMAs that were effective in other models, although the data generated in Figure 1B rely on a strategy that has been shown to be effective for *Salmonella*-mediated inhibition of NOD diabetes onset [29]. It can be seen that in contrast to the earlier studies neither ES-62 nor SMAs 11a and 12b were able to inhibit diabetes onset or to reduce incidence of disease. 

**Figure 2:**
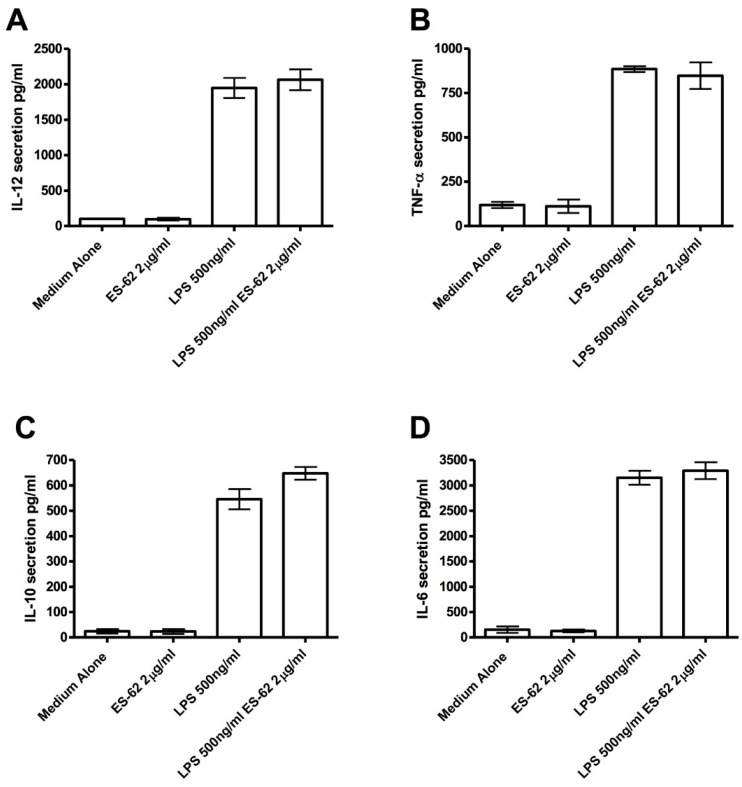
Effects of ES-62 on in vitro cytokine secretion by dendritic cells (DCs). DCs were prepared from bone marrow as described in the Materials and Methods. On day 10, they were harvested and incubated with medium alone, 500 ng/mL of LPS, 2 μg of ES-62 or with both 500 ng/mL of LPS and 2 μg of ES-62. At 72 h, supernatants were harvested and assayed for the presence of (**A**) IL-12p40, (**B**) TNF-α, (**C**) IL-10, and (**D**) IL-6. Data points correspond to the mean plus SEM of each set of samples.

**Figure 3:**
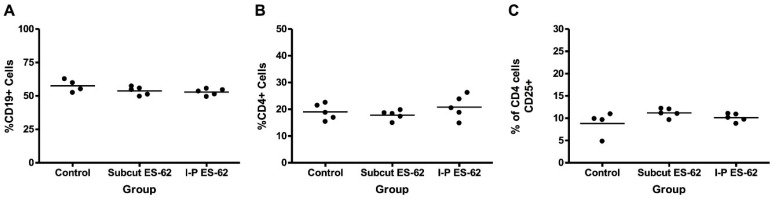
Effects of sub-cutaneous or intra-peritoneal treatment with ES-62 on composition of BDC2.5NOD splenocyte populations. Four-week-old BDC2.5NOD mice were given two injections of 2 μg ES-62 over the course of a week. After one week, splenocytes were harvested, and the percentage of B cells, CD4^+^ cells, and CD4^+^CD25^+^ cells was assessed by staining with (**A**) anti-CD19, (**B**) anti-CD4, and (**C**) anti-CD25, and subsequent FACS analysis. Each dot on the scatter plot corresponds to one individual mouse.

**Figure 4:**
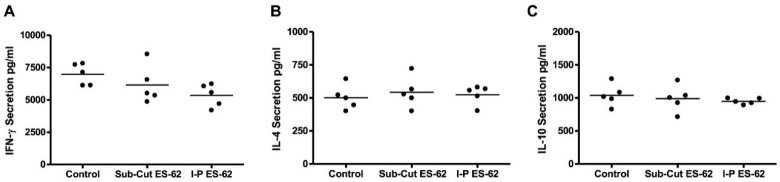
Effects of sub-cutaneous or intra-peritoneal treatment with ES-62 on the ex vivo response of BDC2.5NOD splenocytes to BDC2.5 peptide. Four-week-old BDC2.5NOD mice were given two injections of 2 μg ES-62 over the course of a week. After one week, splenocytes were harvested. Equal numbers of splenocytes from each group were re-stimulated with 1 mg/mL BDC2.5 peptide. Culture supernatants were harvested at 48 h and assessed for the presence of (**A**) IFN-γ, (**B**) IL-4, and (**C**) IL-10 by ELISA. Each dot on the scatter plot corresponds to the average cytokine secretion of splenocytes from one individual mouse.

We also examined the effects of ES-62 on cytokine production by NOD bone marrow derived-DCs (DCs) in vitro, in the presence or absence of LPS. We included LPS in these cultures, as our previous studies had shown that this revealed a cytokine-biasing effect of helminth molecules [14]. There was no significant effect of ES-62 in vitro on cytokine production by NOD DCs (Figure 2). We also employed BDC2.5 mice: these are T cell receptor transgenic mice expressing a T cell receptor for islet antigen on CD4^+^ T cells [26]. We found that the administration of ES-62 to BDC2.5 transgenic mice in vivo had no effect on spleen B and CD4^+^ T cell populations (Figure 3), nor on cytokine production by islet reactive spleen cells (Figure 4).

### 3.2. Multiple Sclerosis

**Figure 5:**
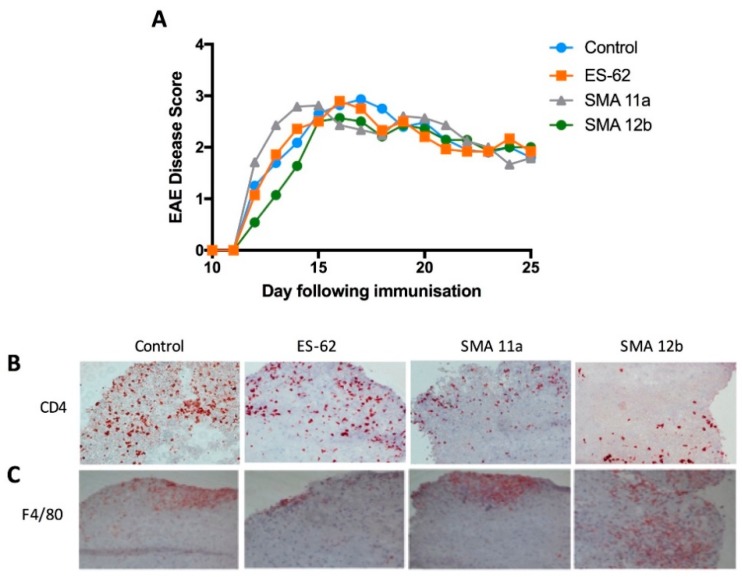
ES-62 and SMAs 11a and 12b do not prevent experimental autoimmune encephalomyelitis. (**A**) Age 7–9 week-old mixed sex C57BL/6 mice were immunised with MOG_35–55_ peptide and treated with PBS (control), ES-62, or SMAs 11a or 12b and clinical disease scores were monitored twice daily (*n* = 5–7). There were no statistically significant differences amongst groups, and hence, SD values have been omitted to ensure the clarity of the figure. Spinal cords were collected from treatment groups and immunohistochemical analysis of CD4 (**B**) or F4/80 (**C**) was performed and representative images are shown. Magnification = 20×.

EAE was induced in mice as described in Materials and Methods. ES-62 and SMAs 11a and 12b were administered to C57BL/6 mice, but no effect was observed on EAE incidence or disease severity (Figure 5A). Immunohistochemical staining with antibodies against CD4 and F4/80 on spinal cord tissues was performed: there was a trend towards fewer infiltrating CD4^+^ cells when administering SMAs (particularly 12b; Figure 5B) but no differences in F4/80^+^ cells (Figure 5C) in the spinal cord between treatment groups. In addition, analysis of the IL-17 and IFN-γ levels in serum and in the conditioned medium of MOG_35–55_-stimulated spleen and lymph node cells was undertaken, but there were no significant differences in the levels of these two cytokines amongst any of the treatment groups (results not shown).

### 3.3. Inflammatory Bowel Disease

**Figure 6:**
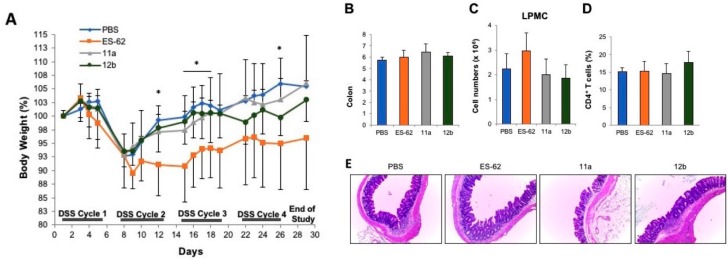
ES-62, and SMAs 11a and 12b do not prevent the induction of chronic colonic inflammation in the DSS-colitis model. (**A**) Body weight changes (as percentage of the initial weight) from the initiation of DSS-colitis was measured during four cycles of DSS with thrice weekly treatments of ES-62 or SMAs (11a or 12b). (**B**) Colon length (cm) was measured at cull. (**C**) Total cell counts and the proportion of CD4^+^ T cells (**D**) present in the lamina propria mononuclear cells (LPMC) were measured. (**E**) Representative Heamatoxylin and Eosin (H & E) staining of colons at time of sacrifice. Magnification = 100×. Data in (**A**–**D**) represent the means ± SD (*n* = 5) and statistical significance as determined by student’s *t* test (PBS versus ES-62) is denoted by * *p* < 0.05.

**Figure 7:**
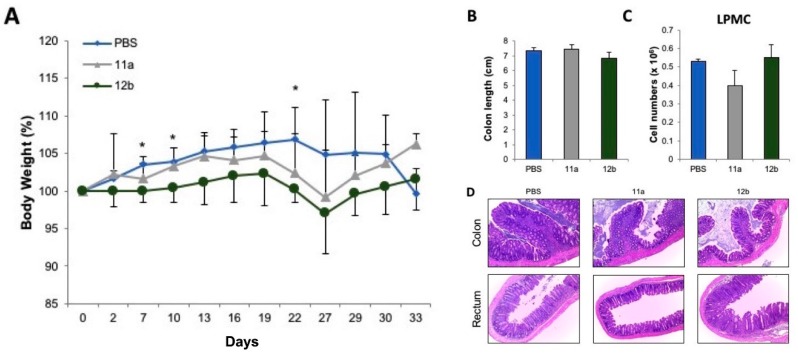
ES-62 SMAs do not attenuate T cell transfer colitis in mice. Naïve CD4^+^ T cells from wild type (WT) mice were injected into *Rag1*^−/−^ mice. PBS or SMAs (11a, 12b) were injected twice per week. (**A**) Body weight changes as a percentage of the initial weight at day 1. (**B**) Colon length. (**C**) Total cell counts in lamina propria mononuclear cells. (**D**) Representative H & E staining of colons at the time of sacrifice. Magnification = 100×. For (**A**–**C**) data represent means ± SD. *N* = 5/group. * *p* < 0.05 as determined by Student’s *t*-test for comparisons between PBS- and 12b-treated mice.

To determine whether ES-62 or SMA 11a and 12b could attenuate inflammatory bowel-like disease, chronic colonic inflammation was administered using four cycles of DSS in drinking water. ES-62, SMA 11a or 12b treatment could not prevent the weight loss observed in mice when their treatment started on day 1 of DSS administration (Figure 6A). In fact, if anything, greater weight loss was observed in ES-62-treated mice compared to PBS-treated mice. Additionally, no significant differences in colon length, cellularity in LPMC, mesenteric lymph nodes (MLN), histology score, and cytokine secretion of re-stimulated LPMC or MLN was observed between ES-62, SMA 11a or 12b, and PBS-treated mice (Figure 6B–E and data not shown). To further characterise any protective effect of SMA 11a or 12b on the development of colitis, a second model of chronic colitis, i.e., the adoptive T cell transfer model was used. Similar to the chronic DSS colitis model, there was no significant differences in weight loss, colon length, cellularity in LPMC, MLN, histology score, and cytokine secretion of restimulated LPMC or MLN between ES-62-, SMA 11a-, 12b- and PBS-treated mice (Figure 7 and data not shown).

These data suggest that SMA 11a, and 12b are not efficient in preventing the development of inflammation in two different models of chronic colitis with different underlying molecular mechanisms of pathogenesis. While chronic DSS colitis depends on initial macrophage recognition of commensal bacteria upon damage of the intestinal layer and subsequent T cell activation, the intestinal inflammation in the adoptive T cell transfer model depends on the proliferation and differentiation of effector T cells in recipient immunocompromised mice lacking Treg cells.

## 4. Discussion

The data presented in this manuscript indicate that ES-62 and its SMAs do not provide any therapeutic benefits in models of T1D, MS and chronic IBD. This is despite the use of treatments based on regimens that are successfully employed in other inflammatory disease models, which apparently involve similar pathogenic mechanisms such as unresolving Th17-driven inflammation. We could have undertaken further experiments employing additional doses of ES-62/SMAs, but our previous experience with ovalbumin-induced airway hypersensitivity suggests that this would have no significant effect on the results [9]. In attempting to understand these failures, the organ-specific nature of the three resistant conditions, targeting pancreatic islets in the NOD mouse, CNS in EAE, and colon in IBD, is a consideration. These organ-specific situations are quite different from the autoimmune disease models (CIA; MRL/Lpr mouse) where ES-62/SMAs have previously shown efficacy, in that the latter have a systemic disease component and exhibit various tissue co-morbidities, reflecting the conditions found in their human disease counterparts. Thus, it is possible that ES-62 and the SMAs might be more suited to tackling pathogenic cellular networks in disorders with a substantial systemic inflammatory component [30,31]. However, at the same time, ES-62/SMAs are very effective in models of asthma that, in being essentially a condition affecting the lungs, could be considered as organ-specific in nature.

Thus, perhaps a more likely factor to consider is the induction of Treg responses, which dominate host immunity involving helminth infections and can also be induced by a number of ES products. Indeed, the induction of such cells by parasitic helminths or their ES products constitutes a protective mechanism in the three ES-62-resistant disease models. For example, SEA-induced Tregs play a role in preventing T1D in NOD mice [15], *H. polygrus*-induced Tregs [32] represent one of the major mechanisms by which this worm is able to prevent colitis in mouse models [33] and infection of mice with *F. hepatica* elicits a (modest) increase in FoxP3^+^ T cells in the pleural cavity, which is associated with increased IL-10 and protection against EAE [19]. Of note therefore, although ES-62 promotes B regulatory responses (Bregs) in models of each of asthma, RA and SLE, [3,5,34], it does not induce Treg responses. Pertinent to this, although protective roles for Bregs have also been proposed for EAE/MS and DSS colitis/IBD, these have generally reflected regulatory mechanisms involving their interaction with Tregs [35]. Focusing on Bregs in particular, it is also worth pointing out that there is a lack of a definitive Breg phenotype(s) and the precise mechanisms (IL-10-dependent and independent) and phenotype of Bregs utilised appears to be disease-dependent. Indeed, as ES-62 appears to restore different Breg subsets in the asthma, RA and SLE models [3,5,34], its potential inability to target the Breg subtypes that are protective in EAE and DSS-colitis may provide a rationale for its failure to afford protection in these models. Likewise, there is a single report of Breg protection against NOD-T1D, the mechanism of which involves TGF-β-production and FasL-mediated apoptosis of auto-reactive T cells [35], but perhaps reflecting the paucity of research in this area, B cell depletion studies in NOD mice and T1D patients suggest that Bregs play a very minor, if any, role in preventing T1D [35]. In any case, despite its ability to restore protective Bregs levels in some inflammatory conditions, its failure to induce Treg-interacting Bregs and also as mentioned earlier, Tregs, may need to be taken into account when considering ES-62’s failure to demonstrate protective effects against disease development in certain models.

Given the role of the gut microbiome in regulating the homeostatic production of inflammation-resolving Bregs in experimental arthritis [36], another potential reason underpinning ES-62-resistance may relate to how the microbiome influences host (auto)immunity in specific conditions, and in particular, the potentially differential impact of ES-62 and helminth infections in these diseases. Certainly, perturbation of the gut microbiota has been shown to influence both ES-62-resistant (diabetes [37], EAE [38] and IBD [39]) and -responsive (asthma [40,41], arthritis [42] and lupus [43]) inflammatory conditions in mice and humans. Perhaps pertinently, certain species of *Clostridium* can promote Tregs, to regulate aberrant inflammation [44,45], and dietary supplementation with probiotics, including *Lactobacillus* species, can reduce IBD and atopic dermatitis by inducing a strong FoxP3^+^CD4^+^ T regulatory response at the site of inflammation [46]. However, although the dramatic differences in microbiota and inflammatory responses observed in animal facilities amongst labs and commercial vendors [47] could contribute to the protective (or not) outcomes of ES-62 treatment in some of the animal models tested, the differential effects of ES-62/SMAs on asthma and EAE were observed within the same animal facility whilst the protective actions of ES-62 on CIA, MRL/Lpr mice, and both acute and chronic asthma models were detected in a range of mouse strains housed in conventional and SPF animal facilities in different geographical locations. Thus, any microbiome-related effects of ES-62 more likely reflect the differential changes in bacterial species in distinct intestinal microenvironments induced under particular pathogenic conditions: for example, changes in the ileum and colon are associated with Th17 and regulatory cell responses, respectively [36,48,49,50]. Thus, it seems likely that analysis of the perturbation of the microbiota and its modulation by ES-62 in resistant and responsive models might provide an important insight into the ability of ES-62 to ameliorate inflammatory conditions, and the protective mechanisms involved.

Finally, it has recently become apparent that the primary effect of ES-62 and the SMAs on immune system cells is to down-regulate the aberrantly elevated MyD88-dependent responses pertaining during chronic inflammation, restoring them back towards the steady-state levels observed in healthy animals. This has been observed with macrophages [6], dendritic cells [51], mast cells [52], and Th17 cells [30], and at least in the case of the SMAs, has been shown to reflect direct interaction with the MyD88 TIR domain [53]. Interestingly, mice lacking MyD88 are resistant to the development of EAE [54] and this may suggest that ES-62/SMAs’ mechanism of action involving reduction rather than the abrogation of adaptor levels [6,30,51,52] may be insufficient to achieve protection against inflammatory responses in this particular model. Alternatively, the reduced My88-dependent signalling in EAE-mice may be compensated for by other inflammatory pathways that are not activated in RA, SLE and asthma models. Clearly, these are ideas that could be explored in future experiments.

In summary, ES-62 and its SMAs generally appear to be effective in treating diseases in model systems that have a strong systemic inflammatory component rather than a more organ-specific pathology. Thus, in the human situation, the SMAs are perhaps more likely to be better suited to treating multifactorial disorders such as RA or SLE which have a range of co-morbidities. Finally, although, ES-62 is somewhat unusual in not inducing Treg responses and this sets it apart to some extent from other helminth-derived molecules, it also re-enforces that ES-62 is not simply a broad immunosuppressant, but rather a focused immunomodulatory agent that can nevertheless treat a variety of conditions.

## References

[1] Maizels R.M., McSorley H.J. (2016). Regulation of the host immune system by helminth parasites. J. Allergy Clin. Immunol..

[2] Pineda M.A., Lumb F., Harnett M.M., Harnett W. (2014). ES-62, a therapeutic anti-inflammatory agent evolved by the filarial nematode *Acanthocheilonema viteae*. Mol. Biochem. Parasitol..

[3] Rodgers D.T., McGrath M.A., Pineda M.A., Al-Riyami L., Rzepecka J., Lumb F., Harnett W., Harnett M.M. (2015). The parasitic worm product, ES-62 targets MyD88-dependent effector mechanisms to suppress ANA production and proteinuria in MRL/Lpr mice. Arthritis Rheumatol..

[4] Rzepecka J., Siebeke I., Coltherd J.C., Kean D.E., Steiger C.N., Al-Riyami L., McSharry C., Harnett M.M., Harnett W. (2013). The helminth product, ES-62, protects against airway inflammation by resetting the Th cell phenotype. Int. J. Parasitol..

[5] Coltherd J.C., Rodgers D.T., Lawrie R.E., Al-Riyami L., Suckling C.J., Harnett W., Harnett M.M. (2016). The parasitic worm-derived immunomodulator, ES-62 and its drug-like small molecule analogues exhibit therapeutic potential in a model of chronic asthma. Sci. Rep..

[6] Al-Riyami L., Pineda M.A., Rzepecka J., Huggan J.K., Khalaf A.I., Suckling C.J., Scott F.J., Rodgers D.T., Harnett M.M., Harnett W. (2013). Designing anti-inflammatory drugs from parasitic worms: A synthetic small molecule analogue of the Acanthocheilonema viteae product ES-62 prevents development of collagen-induced arthritis. J. Med. Chem..

[7] Al-Riyami L., Rodgers D., Rzepecka J., Pineda M.A., Suckling C.J., Harnett M.M., Harnett W. (2015). Protective effect of small molecule analogues of the Acanthocheilonema viteae secreted product ES-62 on oxazolone-induced ear inflammation. Exp. Parasitol..

[8] Janicova L., Rzepecka J., Rodgers D.T., Doonan J., Bell K.S., Lumb F.E., Suckling C.J., Harnett M.M., Harnett W. (2016). Testing small molecule analogues of the Acanthocheilonema viteae immunomodulator ES-62 against clinically relevant allergens. Parasite Immunol..

[9] Rzepecka J., Coates M.L., Saggar M., Al-Riyami L., Coltherd J., Tay H.K., Huggan J.K., Janicova L., Khalaf A.I., Siebeke I. (2014). Small molecule analogues of the immunomodulatory parasitic helminth product ES-62 have anti-allergy properties. Int. J. Parasitol..

[10] Rzepecka J., Pineda M.A., Al-Riyami L., Rodgers D.T., Huggan J.K., Lumb F.E., Khalaf A.I., Meakin P.J., Corbet M., Ashford M.L. (2015). Prophylactic and therapeutic treatment with a synthetic analogue of a parasitic worm product prevents experimental arthritis and inhibits IL-1β production via NRF2-mediated counter-regulation of the inflammasome. J. Autoimmun..

[11] Lumb F.E., Doonan J., Bell K.S., Pineda M.A., Corbet M., Suckling C.J., Harnett M.M., Harnett W. (2017). Dendritic cells provide a therapeutic target for synthetic small molecule analogues of the parasitic worm product, ES-62. Sci. Rep..

[12] Doonan J., Lumb F.E., Pineda M.A., Tarafdar A., Crowe J., Khan A.M., Suckling C.J., Harnett M.M., Harnett W. (2018). Protection Against Arthritis by the Parasitic Worm Product ES-62, and Its Drug-Like Small Molecule Analogues, Is Associated with Inhibition of Osteoclastogenesis. Front. Immunol..

[13] Rzepecka J., Harnett W., Bruschi F. (2014). Can the study of helminths be fruitful for human diseases?. Helminth Infections and Their Impact on Global Public Health.

[14] Zaccone P., Fehérvári Z., Jones F.M., Sidobre S., Kronenberg M., Dunne D.W., Cooke A. (2003). *Schistosoma mansoni* antigens modulate the activity of the innate immune response and prevent onset of type 1 diabetes. Eur. J. Immunol..

[15] Zaccone P., Burton O., Miller N., Jones F.M., Dunne D.W., Cooke A. (2009). *Schistosoma mansoni* egg antigens induce Treg that participate in diabetes prevention in NOD mice. Eur. J. Immunol..

[16] Lund M.E., O’Brien B.A., Hutchinson A.T., Robinson M.W., Simpson A.M., Dalton J.P., Donnelly S. (2014). Secreted Proteins from the Helminth Fasciola hepatica Inhibit the Initiation of Autoreactive T Cell Responses and Prevent Diabetes in the NOD Mouse. PLoS ONE.

[17] Hübner M.P., Stocker J.T., Mitre E. (2009). Inhibition of type 1 diabetes in filaria-infected non-obese diabetic mice is associated with a T helper type 2 shift and induction of FoxP3^+^ regulatory T cells. Immunology.

[18] Sewell D., Qing Z., Reinke E., Elliot D., Weinstock J., Sandor M., Fabry Z. (2003). Immunomodulation of experimental autoimmune encephalomyelitis by helminth ova immunization. Int. Immunol..

[19] Zheng X., Hu X., Zhou G., Lu Z., Qiu W., Bao J., Dai Y. (2008). Soluble egg antigen from Schistosoma japonicum modulates the progression of chronic progressive experimental autoimmune encephalomyelitis via Th2-shift response. J. Neuroimmunol..

[20] Walsh K.P., Brady M.T., Finlay C.M., Boon L., Mills K.H.G. (2009). Infection with a Helminth Parasite Attenuates Autoimmunity through TGF-β-Mediated Suppression of Th17 and Th1 Responses. J. Immunol..

[21] Donskow-Łysoniewska K., Krawczak K., Doligalska M. (2012). Heligmosomoides polygyrus: EAE remission is correlated with different systemic cytokine profiles provoked by L4 and adult nematodes. Exp. Parasitol..

[22] Wilson M.S., Taylor M.D., O’Gorman M.T., Balic A., Barr T.A., Filbey K., Anderton S.M., Maizels R.M. (2010). Helminth-induced CD19+CD23hi B cells modulate experimental allergic and autoimmune inflammation. Eur. J. Immunol..

[23] Hasby E.A., Hasby Saad M.A., Shohieb Z., El Noby K. (2015). FoxP3+ T regulatory cells and immunomodulation after *Schistosoma mansoni* egg antigen immunization in experimental model of inflammatory bowel disease. Cell. Immunol..

[24] Ferreira I., Smyth D., Gaze S., Aziz A., Giacomin P., Ruyssers N., Artis D., Laha T., Navarro S., Loukas A. (2013). Hookworm Excretory/Secretory Products Induce Interleukin-4 (IL-4)^+^IL-10^+^ CD4^+^ T Cell Responses and Suppress Pathology in a Mouse Model of Colitis. Infect. Immun..

[25] Wilson E.H., Deehan M.R., Katz E., Brown K.S., Houston K.M., O’Grady J., Harnett M.M., Harnett W. (2003). Hyporesponsiveness of murine B lymphocytes exposed to the filarial nematode secreted product ES-62 in vivo. Immunology.

[26] Katz J.D., Wang B., Haskins K., Benoist C., Mathis D. (1993). Following a dibetogenic T cell from genesis through pathogenesis. Cell.

[27] Takedatsu H., Michelsen K.S., Wei B., Landers C.J., Thomas L.S., Dhall D., Braun J., Targan S.R. (2008). TL1A (TNFS15) regulates the development of chronic colitis by modulating both T-helper and T-helper 17 activation. Gastroenterology.

[28] Weigmann B., Tubbe I., Seidel D., Nicolaev A., Bewcker C., Neurath M.F. (2007). Isolation and subsequent analysis of murine lamina propria mononuclear cells from colonic tissue. Nat. Protocol..

[29] Zaccone P., RAine T., Sidobre S., Kronenberg M., Mastroeni P., Cooke A. (2004). *Salmonella typhimurium* infection halts development of type 1 diabetes in NOD mice. Eur. J. Immunol..

[30] Pineda M.A., McGrath M.A., Smith P.C., Al-Riyami L., Rzepecka J., Gracie J.A., Harnett W., Harnett M.M. (2012). The parasitic helminth product ES-62 suppresses pathogenesis in collagen-induced arthritis by targeting the interleukin-17-producing cellular network at multiple sites. Arthritis Rheum..

[31] Pineda M.A., Rodgers D.T., Al-Riyami L., Harnett W., Harnett M.M. (2014). ES-62 protects against collagen-induced arthritis by resetting interleukin-22 toward resolution of inflammation in the joints. Arthritis Rheumatol. Hoboken.

[32] Finney C.A.M., Taylor M.D., Wilson M.S., Maizels R.M. (2007). Expansion and activation of CD4^+^CD25^+^ regulatory T cells in *Heligmosomoides polygyrus* infection. Eur. J. Immunol..

[33] Hang L., Blum A.M., Setiawan T., Urban J.P., Stoyanoff K.M., Weinstock J.V. (2013). *Heligmosomoides polygyrus* bakeri infection activates colonic Foxp3^+^ T cells enhancing their capacity to prevent colitis. J. Immunol. Baltim..

[34] Rodgers D.T., Pineda M.A., McGrath M.A., Al-Riyami L., Harnett W., Harnett M.M. (2013). Protection against collagen-induced arthritis in mice afforded by the parasitic worm product ES-62 is associated with restoration of the levels of interleukin 10-producing B cells and reduced plasma cell infiltration of the joints. Immunology.

[35] Ray A., Dittel B.N. (2017). Mechanisms of regulatory B cell function in autoimmune and inflammatory diseases beyond IL-10. J. Clin. Med..

[36] Rosser E.C., Oleinika K., Tonon S., Doyle R., Bosma A., Carter N.A., Harris K.A., Jones S.A., Klein N., Mauri C. (2014). Cells are induced by gut microbiota-driven interleukin-1β and interleukin-6 production. Nat. Med..

[37] Burrows M.P., Volchkov P., Kobayashi K.S., Chervonsky A.V. (2015). Microbiota regulates type 1 diabetes through Toll-like receptors. Proc. Natl. Acad. Sci. USA.

[38] Ochoa-Repáraz J., Mielcarz D.W., Wang Y., Begum-Haque S., Dasgupta S., Kasper D.L., Kasper L.H. (2010). A polysaccharide from the human commensal *Bacteroides fragilis* protects against CNS demyelinating disease. Mucosal Immunol..

[39] Li M., Wu Y., Hu Y., Zhao L., Zhang C. (2017). Initial gut microbiota structure affects sensitivity to DSS-induced colitis in a mouse model. Sci. China Life Sci..

[40] Smits H.H., Hiemstra P.S., Prazeres da Costa C., Ege M., Edwards M., Garn H., Howarth P.H., Jartti T., de Jong E.C., Maizels R.M. (2016). Microbes and asthma: Opportunities for intervention. J. Allergy Clin. Immunol..

[41] Kyburz A., Muller A. (2016). The Gastrointestinal Tract Microbiota and Allergic Diseases. Dig. Dis..

[42] Clemente J.C., Manasson J., Scher J.U. (2018). The role of the gut microbiome in systemic inflammatory disease. BMJ.

[43] Abdollahi-Roodsaz S., Abramson S.B., Scher J.U. (2016). The metabolic role of the gut microbiota in health and rheumatic disease: Mechanisms and interventions. Nat. Rev. Rheumatol..

[44] Atarashi K., Tanoue T., Oshima K., Suda W., Nagano Y., Nishikawa H., Fukuda S., Saito T., Narushima S., Hase K. (2013). T_reg_ induction by a rationally selected mixture of Clostridia strains from the human microbiota. Nature.

[45] Atarashi K., Tanoue T., Shima T., Imaoka A., Kuwahara T., Momose Y., Cheng G., Yamasaki S., Saito T., Ohba Y. (2011). Induction of Colonic Regulatory T Cells by Indigenous Clostridium Species. Science.

[46] Kwon H.-K., Lee C.-G., So J.-S., Chae C.-S., Hwang J.-S., Sahoo A., Nam J.H., Rhee J.H., Hwang K.-C., Im S.-H. (2010). Generation of regulatory dendritic cells and CD4^+^Foxp3^+^ T cells by probiotics administration suppresses immune disorders. Proc. Natl. Acad. Sci. USA.

[47] Ericsson A.C., Davis J.W., Spollen W., Bivens N., Givan S., Hagan C.E., McIntosh M., Franklin C.L. (2015). Effects of Vendor and Genetic Background on the Composition of the Fecal Microbiota of Inbred Mice. PLoS ONE.

[48] Donaldson G.P., Lee S.M., Mazmanian S.K. (2016). Gut biogeography of the bacteria microbiota. Nat. Rev. Microbiol..

[49] Grigg J.B., Sonnenberg G.F. (2017). Host-Microbiota Interactions Shape Local and Systemic Inflammatory Diseases. J. Immunol..

[50] Wu H.J., Ivanov I.I., Darce J., Hattori K., Shima T., Umesaki Y., Littman D.R., Benoist C., Mathis D. (2010). Gut-residing segmented filamentous bacteria drive autoimmune arthritis via T helper 17 cells. Immunity.

[51] Ball D.H., Tay H.K., Bell K.S., Coates M.L., Al-Riyami L., Rzepecka J., Harnett W., Harnett M.M. (2013). Mast cell subsets and their functional modulation by the *Acanthocheilonema viteae* product ES-62. J. Parasitol. Res..

[52] Eason R.J., Bell K.S., Marshall F.A., Rodgers D.T., Pineda M.A., Steiger C.N., Al-Riyami L., Harnett W., Harnett M.M. (2016). The helminth product ES-62 modulates dendritic cell responses by inducing the selective autophagolysosomal degradation of TLR transducers, as exemplified by PKCδ. Sci. Rep..

[53] Suckling C.J., Alam S., Olson M.A., Saikh K.U., Harnett M.M., Harnett W. (2018). Small molecule analogues of the parasitic worm product ES-62 interact with the TIR domain of MyD88 to inhibit pro-inflammatory signaling. Sci. Rep..

[54] Marta M., Andersson A., Isaksson M., Kampe O., Lobell A. (2008). Unexpected regulatory roles of TLR4 and TLR9 in experimental autoimmune encephalomyelitis. Eur. J. Immunol..

